# Improving anaemia diagnosis using peripheral blood smear with remote interpretation in adults living with HIV with moderate to severe anaemia: A prospective study nested within the Kilombero and Ulanga antiretroviral cohort

**DOI:** 10.1371/journal.pone.0293084

**Published:** 2023-10-19

**Authors:** Vanesa Anton-Vazquez, Dorcas Mnzava, James Okuma, Slyakus Mlembe, Laura Lo Riso, Jose Maria Sanchez, Robert Ndege, Aneth Vedastus Kalinjuma, Namvua Kimera, Anna Eichenberger, Giovanni Jacopo Nicoletti, Herieth Wilson, Fiona Vanobberghen, Maja Weisser, on behalf of the KIULARCO Study Group

**Affiliations:** 1 Ifakara Health Institute, Ifakara, Tanzania, United Republic of Tanzania; 2 Swiss Tropical & Public Health Institute, Allschwil, Switzerland; 3 University of Basel, Basel, Switzerland; 4 Department of Haematology, Son Espases University Hospital, Palma de Mallorca, Spain; 5 St. Francis Referral Hospital, Ifakara, Tanzania, United Republic of Tanzania; 6 Department of Epidemiology and Biostatistics, Faculty of Health Sciences, School of Public Health, University of the Witwatersrand, Johannesburg, South Africa; 7 Department of Infectious Diseases, University Hospital Bern, Bern, Switzerland; 8 Division of Infectious Diseases & Hospital Epidemiology, University Hospital Basel, Basel, Switzerland; University of Cape Town Faculty of Science, SOUTH AFRICA

## Abstract

**Introduction:**

In low-resource settings, anaemia is a very common condition. Identification of anaemia aetiologies remains challenging due to the lack of diagnostic tools and expertise. We aimed to improve anaemia diagnostics using peripheral blood smear (PBS) with remote interpretation in people living with HIV (PLHIV) with moderate to severe anaemia.

**Methods:**

We conducted a prospective study nested within the Kilombero and Ulanga Antiretroviral Cohort, including non-pregnant PLHIV aged ≥18 years presenting with moderate (haemoglobin 7.0–9.9 g/dl) or severe (<7.0 g/dl) anaemia at any visit from January 2019 to December 2020. For each participant, ten PBS images, full blood count and clinical details were shared with a haematologist for remote interpretation (enhanced care). Identification of anaemia etiologies and potential impact on treatment was compared between enhanced and standard care.

**Results:**

Among 400 PLHIV with moderate to severe anaemia, 349 (87%) were female, median age was 40 years (interquartile range (IQR) 35–46)), 65 (17%) had a body mass index <18.5 kg/m^2^, 215 (54%) had HIV WHO stage III/IV, 79 (20%) had a CD4 cell count <200 cells/μl and 317 (89%) had HIV viral load <100 copies/ml. Severe anaemia was diagnosed in 84 (21%). Suspected multiple aetiologies were documented more frequently by enhanced care compared to standard care 267 (67%) vs 20 (5%); p<0.001. Suspected iron deficiency was the most frequent aetiology (n = 337; 84%), followed by chronic disease (n = 199; 50%), folate/vitamin B12 deficiency (n = 78; 20%) and haemoglobinopathy (n = 83; 21%). In 272 participants (68%), enhanced care revealed additional clinically relevant findings with impact on the treatment recommendation.

**Conclusion:**

Remote interpretation of PBS combined with clinical information and blood cell count results can provide insights to the suspected aetiological diagnosis of moderate and severe anaemia in rural low-resource settings and impact specific treatment.

## Introduction

Anaemia is the most common haematologic disorder among people living with HIV (PLHIV), affecting 20–84% worldwide [1, 2]. Anaemia is associated with HIV progression[3–5] and mortality [6–10]. In sub-Saharan Africa, where anaemia is a major public health problem in the general population, the anaemia prevalence in PLHIV reaches to up to 75–87% [11, 12] depending on the different geo-socio-economic backgrounds [13–16]. Despite the introduction of antiretroviral therapy (ART), the prevalence of anaemia in adults living with HIV ranges between 58–71% [6, 7, 17, 18]. The causes of anaemia among PLHIV are multifactorial and many of them may co-exist, for instance, myelosuppression by HIV [8] or antiretroviral medication such as zidovudine [9], chronic diseases, blood loss or ineffective production of red blood cells due to nutritional deficiencies such as iron, folic acid or vitamin B12 [10, 11]. In addition, co-infections with a bacterial, mycobacterial, viral, parasitic and fungal organisms can disrupt normal haematopoiesis and contribute to the development of anaemia. In sub-Saharan Africa, the overlap between tuberculosis, malaria and helminths among PLHIV is responsible for the greatest burden of anaemia in this setting [19–21].

Accurate characterisation of anaemia is key to understanding the aetiology and guiding treatment [7]. In clinical practice, the aetiological diagnosis of anaemia depends on a “framework approach”, which is based on clinical information and laboratory test results. The latter include a wide range of costly and sophisticated haematology and biochemistry analysis [22]. In low-resource settings (LRS), the limited availability of diagnostic tests for anaemia poses a challenge in accurately interpreting and effectively managing anaemia, particularly in cases where multiple causes may be involved [23, 24]. An important tool in these settings is the peripheral blood smear (PBS), a laboratory technique used for the identification of morphological abnormalities in red blood cells. The PBS, a relatively affordable and practical diagnostic tool, allows for the detection of various abnormalities that can provide crucial insights into the underlying causes of anaemia [25]. Expert interpretation of the PBS by a haematologist can assist clinicians in patient management and a reduction of unnecessary blood transfusions [25–27]. However, PBS is not widely available in LRS as its interpretation requires the expertise of a trained professional, which can be labour-intensive and time-consuming [25]. In this respect, telemedicine has evolved as a convenient opportunity for optimising healthcare for various purposes such as specialist interpretation of imaging technologies and advising on treatment choice. Tele-haematology allows remote interpretation of blood smears by experienced haematologists for LRS with limited trained healthcare staff [25].

In this study, we aimed to improve anaemia diagnostics using PBS with remote interpretation in PLHIV with moderate to severe anaemia.

## Methods

### Study design and setting

We conducted a prospective study within the Kilombero and Ulanga Antiretroviral Cohort (KIULARCO) [28]. KIULARCO is a prospective cohort of patients seen at the Chronic Diseases Clinic Ifakara, the HIV care and treatment centre of the St. Francis Referral Hospital in Ifakara, located in rural Tanzania. KIULARCO study procedures have been described elsewhere [28, 29]. In brief, after signing an informed consent for KIULARCO participation at enrolment, socio-demographic, clinical and laboratory data are collected from routine care and entered into an electronic medical record system.

### Participants

In this study, we prospectively included PLHIV aged ≥18 years enrolled in KIULARCO, and who presented with moderate (haemoglobin (Hb) 7.0–9.9 g/dl) or severe (Hb <7.0 g/dl) anaemia according to the Demographic and Health Surveys (DHS) [30]. Enrolment into this study was done at KIULARCO baseline or at any follow-up in- or outpatient visit from January 2019 to December 2020, with follow- up through October 2021. We excluded pregnant women, children, participants who received a blood transfusion in the last month and those who declined KIULARCO participation.

### Study workflow and procedures in the clinics

Within KIULARCO, all participants receive an automated full blood count at enrolment, after six months and thereafter yearly, if clinically stable or upon clinical indication. For this study, the laboratory staff informed the clinician of a patient with a moderate or severe anaemia based upon a routine full blood count. If insufficient blood was available, a second blood withdrawal was performed on the same or the next day for the performance of PBS. Ten digital microscopic images of each PBS were sent together with results from the automated blood analyser and anonymized patient details via a secured online platform to the haematologist. Upon diagnosis of anaemia—usually the same day -, the clinician started treatment based on the clinical history and full blood count results (standard of care). Within the following 2 weeks, the remote haematologist interpreted the PBS images and blood analyser results and provided a written report with the suspected aetiology of anaemia and the recommended treatment (enhanced care). Patients with moderate anaemia were scheduled as per standard routine follow-up, whereas those with severe anaemia were scheduled within two weeks. During each follow-up appointment, the responsible clinician had access to the treatment recommendations provided by the haematologist and management was modified accordingly (**S1 Fig).** Suspected anaemia aetiologies and suggested treatment recommendations were categorised according to a predefined set of diagnoses and treatments (**Table 1).**

**Table 1:** Suspected aetiologies of anaemia based on enhanced care^a^ and standard care^b^.

	Enhanced care				
	Standard care			Blood smear findings	Suggested treatment
Suspected aetiologies	Clinical details	Blood analyser ^c^			
		MCV	MCH		
**Iron deficiency**	Bleeding, evidence of helminth infection	Decreased	Decreased	Microcytosis (red cells smaller than ± 7 μm in diameter), hypochromia (red blood cells have an expanded central zone of pallor greater than one-third of the diameter of the cell), anisocytosis and poikilocytosis, elliptocytes, dianocytes.	Iron replacement
**Chronic disease**	Underlying chronic disease (kidney/liver failure, malignancy, infection)	Decreased/ Normal	Normal/ Decreased	Normochromia (red blood cell with a normal amount of colour within the red blood cell), normocytosis or microcytosis, anisocytosis and poikilocytosis, echinocytes (if anaemia related with renal or hepatic failure), acanthocytes.	Treatment of the underlying condition (eg. Infection, malignancy)
**Folate/ vitaminB12 deficiency**	Malabsorption signs, glossitis, paresthesia, ataxia, neuropathy	Increased	Increased/ Normal	Macrocytosis (red cells are larger than 9 μm in diameter), hyperchromia or normochromia, macro-ovalocytes, hypersegmentation of neutrophils.	Oral folates and intramuscular vitamin B12
**Haemoglobinopathy**	Long-term anaemia. Pain crisis, splenomegaly	Decreased	Normal/ Decreased	Thalassaemia trait: Hypochromia, microcytosis, dianocytes, polychromasia, basophilic stippling inclusions, anisopoikilocytosis, elliptocytes. Sickle cell disease: Normocytosis or microcytosis, normochromia, sickled cells, schistocytes, helmet-red cells, anisopoikilocytosis, polychromasia. Spherocytosis: normocytosis or microcytosis, hyperchromia, spherocytes, polychromasia. Haemoglobin C disease: microcytosis, hypochromia, target cells, intracellular crystals.	Oral folates
**Other (bone marrow toxicity, haemolysis)**	Zidovudine, Cotrimoxazole. Jaundice, hepato-splenomegaly	Normal/ Decreased	Normal/ Decreased	Bone marrow toxicity: Macrocytosis, hyperchromia or normochromia, round macrocytes, acanthocytes, stomatocytes, knizocytes. Haemolysis: Normocytosis, normochromia, schistocytes, helmet-red cells, microspherocytes, anisopoikylocytosis, rouleaux, polychromasia.	Underlying condition. Discontinuation of haemato-toxic treatment

^a^ haematology expert interpretation of blood analyser results, clinical details and peripheral blood smear findings

^b^ clinician’s interpretation of blood analyser results and clinical details

^c^ Results provided by the blood analyser: Mean corpuscular volume (MCV): MCV< 80 fL (decreased); MCV 80–100 fL (normal); MCV > 100 fL (increased). Mean corpuscular haemoglobin (MCH): MCH < 27 pg (decreased); MCH 27–32 pg (normal); MCH >32 pg (increased). This table was generated using different literature sources [38, 45–50].

### Laboratory examinations

PBSs were prepared in the laboratory for cell morphology observation following the European Quality Assurance in Laboratory Medicine (EQALM) Haematology Working Group Guidelines [31]. A static imaging system was used, AmScope—1.3 MP USB 2.0 Digital Microscope Camera with Measuring Imaging Software—MU130. Ten microscopic images from different PBS fields, which were representative of the whole slide, were captured manually and stored with a digital camera attached to a microscope by a trained laboratory technician. Digital images were taken using high optical magnification x 100 (oil). Subsequently, the images were transferred via a secured online platform for interpretation. Cut-off values for grading of RBC morphological abnormalities were based on consensus guidelines [32]. The automated haematology analyser used for full blood count was Sysmex XP-300 or Sysmex KX-21N (Sysmex Europe SE, Germany). Blood analyser results shared with the haematologists included haemoglobin (Hb), white blood cell count (WBC), platelet count, monocytes-basophils-eosinophils mixed (MXD), mean corpuscular volume (MCV), mean corpuscular haemoglobin (MCH) and haematocrit (HCT). A rapid malaria diagnostic test was performed in all participants. Additional investigations to exclude and/or to confirm specific causes of anaemia, such as tests for iron level, ferritin, vitamin B12, folates, reticulocytes, TSH or LDH could not be performed due to the lack of resources and diagnostic testing availability in our setting. Reports were generated within two weeks of PBS performance. In cases of severe anaemia (Hb <7 g/dl), hospital admission or clinical deterioration, the time to report to the clinician by the haematology team was usually under 24 hours and always within 48 hours. In case of detection of bacteria/fungi, clinicians were contacted in order to initiate treatment immediately.

### Accuracy, reliability and quality assurance of PBS images

Peripheral blood smear images for each case were reviewed by 2 haematologists independently and a consensus was reached through joint review of the case, considering patient’s clinical history and full blood count. The first twenty glass slides prepared by a laboratory technician on site with specific training in peripheral blood smear review were compared to PBS images with an acceptable comparability as documented in the literature [33]. Before interpretation, quality parameters including image focus, resolution, magnification, staining and image artifacts were assessed to ensure accurate and reliable interpretation of RBC morphology [34]. Acceptable and good quality images were directly interpreted, whereas re-staining and/or repeat images were performed on site upon request when the quality was deemed to be poor.

### Definitions

We defined a set of five aetiological groups of anaemia based on the erythrocyte shape, size, haemoglobinisation and specific morphologic characteristics evaluated by light microscopic examination of the blood film and by measurement of the MCV and MCH results of the automated blood analyser [35]. The five suspected aetiologies were iron deficiency anaemia, chronic disease type, folate and/or vitamin B12 deficiency, haemoglobinopathy and other (suspected haemolysis or bone marrow disease). Aetiologic treatment included supplemental iron, folate and B12 replacement, treatment of the underlying condition and discontinuation of a haematotoxic drug (**Table 1**). Nutritional status was defined by measurement of the body mass index (BMI), whereby underweight was defined as a BMI <18.5 kg/m^2^, normal weight as a BMI of 18.5–24 kg/m^2^, overweight as BMI of 25–29 kg/m^2^, and obesity as BMI ≥30 kg/m^2^. Comorbidities (hypertension, renal impairment, chronic heart disease, chronic liver disease, diabetes mellitus and oncological disease) were captured prospectively based on the International Statistical Classification of Diseases and Related Health Problems 10^th^ Revision (ICD-10) [36]. Renal impairment was determined using the CKD-Epid formula to calculate the glomerular filtration rate (eGFR). An eGFR of <60 mL/min/1.73m2 was defined as renal impairment. Other chronic conditions such as chronic liver disease or malignancy were not routinely confirmed in our center due to lack of resources, however some cases were confirmed in tertiary centres using imaging and or histopathology testing. Clinical diagnoses for co-infections were done by ICD-10 codes at the time of enrolment, not all diagnoses on co-infections were microbiologically confirmed. For malaria a rapid test or blood smear was done. Microscopic stool examination was done by a trained laboratory technician. Toxoplasmosis in HIV was a clinical diagnosis and Tuberculosis could be diagnosed either clinically or be confirmed by Xpert TB/RIF®. Bacterial infections were mostly diagnosed clinically. Cryptococcal infections were confirmed by antigen testing, candida infections were diagnosed clinically. Chronic hepatitis B was diagnosed by HbS Antigen test and Syphilis by a VDRL test.

Potential added clinical value was defined as when the PBS interpretation included a morphological abnormality that had the potential to impact patient management.

### Data collection and variables

We collected participants’ data from the KIULARCO electronic medical record system (OpenMRS). Details on anaemia aetiology, recommended treatment and clinical outcomes were prospectively collected in a purpose-built anonymised database. The following variables were assessed at the time of inclusion into the anaemia study: socio-demographics (age, gender, marital status, highest education level, occupation and distance of residence to the clinic), clinical (BMI, ART, HIV WHO stage, CD4 cell counts, viral suppression, year since ART started, comorbidities and co-infections) and laboratory (Hb, WBC, platelet count, MXD, MCV, MCH and HCT from the blood analyser). Encrypted data were stored on a secured local database and an online platform (SWITCH drive) until analysis.

### Study outcomes

The primary outcome was to identify and describe the suspected aetiologies of moderate and severe anaemia in PLHIV provided by enhanced versus the standard of care. Secondary outcomes included describing the suggested treatment recommendations for moderate and severe anaemia according to enhanced or standard of care and identifying the potential added clinical value of PBS interpretation. For comparison, the same set of pre-defined anaemia aetiologies and recommended treatment was used (**Table 1**). A PBS review was classified as having potential added clinical value if the final interpretation met the following criteria: (i) a morphologic diagnostic finding was detected by microscopy which could not be diagnosed by automated blood cell analyser alone and (ii) the overall interpretation was likely to impact patient management. For instance, a finding of dimorphic red blood cell population alongside with other specific morphological features suggestive of a dual aetiology of anaemia, or findings consistent with haemoglobinopathy or potential haemato-oncology disorder, or any finding of microorganisms were considered to have potential added clinical value.

### Statistical analysis

Prospectively collected data extracted from KIULARCO OpenMRS and a purpose-built anonymised database were merged and analysed using Stata 16. Demographic characteristics were summarized using medians and interquartile ranges (IQR) for continuous variables and frequencies and percentages for categorical variables. The distribution of suspected aetiologies of anaemia and recommended treatment based on enhanced care, and the proportion of treatment decisions in severe and moderate anaemia were compared using McNemar test. Comparison of PBS characteristics between moderate and severe anaemia groups was analysed descriptively.

### Ethical approval

Written informed consent was obtained from all participants, as part of the KIULARCO enrolment, for inclusion in the study. Ethical approval for KIULARCO was obtained from the Ifakara Health Institute review board (IHI/IRB/No16-2006) and the National Health Research Committee of the National Institute of Medical Research of Tanzania (NIMR/HQ/R.8a/Vol.IX/620) with yearly renewal.

## Results

**Fig 1:**
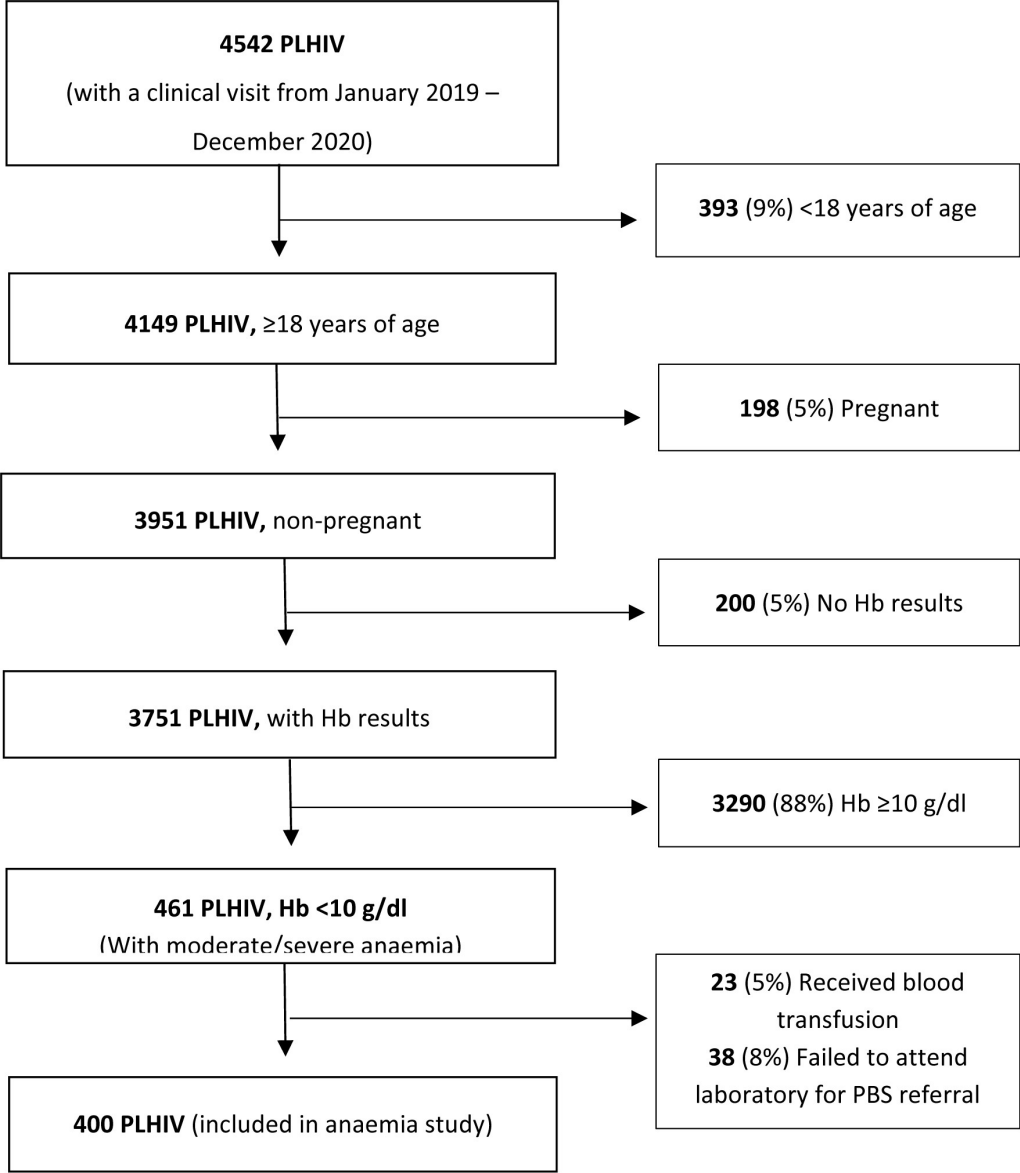
Flow diagram showing the number of participants included in the analysis.

A total of 4542 PLHIV were screened for study eligibility. Of these, 4142 participants were excluded: 393 (10%) were aged <18 years, 198 (5%) were pregnant, 200 (5%) had no Hb measurement, 3290 (79%) had Hb ≥10.0 g/dl and 61 (2%) had a prior blood transfusion or failed to attend laboratory for PBS referral. The remaining 400 PLHIV with a confirmed moderate or severe anaemia were included in the main analysis (**Fig 1**).

### Baseline characteristics

Of 400 participants, 349 (87%) were female; the median age was 40 years (IQR 35–46), 65 (17%) were underweight, 215 (54%) had an HIV WHO stage III/IV, 79 (20%) a CD4 cell count <200/μl, 317 (89%) had HIV viral load <100 copies/ml (**Table 2**). The majority of participants (n = 375; 94%) were on ART and 265 (71%) had been on ART for 5 years or more. The most common ART combination was a non-AZT- containing regimen (n = 350; 93%). Co-morbidities were present in 145 (36%), with co-infection (n = 101; 25%) being the most frequent, including bacterial origin (n = 28; 7%), mycobacteria (n = 26; 7%) and parasitic (n = 20; 5%), followed by arterial hypertension (n = 86; 22%) and chronic kidney disease (n = 46; 12%). Normocytosis by MCV was present in 146 (37%) and 327 (82%) had hypochromia by MCH. Among the 84 participants with severe anaemia, a higher percentage lived far from the clinic compared to those with moderate anaemia (29 (35%) versus 88 (28%), respectively). Additionally, a greater proportion of the severely anaemic participants were underweighted (18 (22%) versus 47 (15%); p = 0.181), were not on ART (10 (12%) versus 15 (5%); p = 0.023) and had an advanced HIV stage (59 (70%) versus 156 (49%); p = 0.002) compared to those with moderate anaemia.

**Table 2:** Baseline^a^ characteristics of study participants.

Patient characteristics	All	Moderate anaemia	Severe anaemia
	N = 400	N = 316	N = 84
**Socio-demographics**			
** Age (years), median (IQR)**	40 (35–46)	40 (34–47)	41 (37–46)
** Gender, Female n (%)**	349 (87%)	275 (87%)	74 (88%)
** Married Status, n (%)**			
** Married/Cohabiting**	223 (63%)	172 (55%)	51 (61%)
** Never married**	45 (11%)	36 (12%)	9 (11%)
** Separated/divorced/widowed**	128 (32%)	106 (34%)	22 (27%)
** Education, n (%)**			
** None**	42 (11%)	32 (10%)	10 (12%)
** Primary**	330 (83%)	264 (84%)	66 (79%)
** Secondary and above/other**	26 (7%)	17 (5%)	9 (10%)
** Occupation, Farmer, n (%)**	339 (85%)	264 (84%)	75 (89%)
** Distance of home to clinic, km, n (%)**			
** ≤1 Km**	197 (49%)	159 (51%)	38 (45%)
** 2 –<50 Km**	86 (22%)	69 (22%)	17 (20%)
** ≥50 Km**	117 (29%)	88 (28%)	29 (35%)
**Clinical parameters**			
Body Mass Index (BMI), Kg/m^2^, n (%)			
** Underweight (BMI <18.5)**	65 (17%)	47 (15%)	18 (22%)
** Normal, (BMI 18.5 - <25)**	220 (56%)	168 (55%)	52 (63%)
** Overweight, (BMI 25 - <30)**	80 (21%)	73 (24%)	7 (8%)
** Obese, (BMI ≥30)**	25 (6%)	21 (7%)	4 (5%)
** ART Regimen, n (%)**			
** No ART**	25 (6%)	15 (5%)	10 (12%)
** AZT-containing**	25 (6%)	21 (7%)	4 (5%)
** Non-AZT-containing**	350 (88%)	280 (89%)	70 (83%)
** WHO Stage, n (%)**			
** I/II**	185 (46%)	160 51%)	25 (30%)
** III/IV**	215 (54%)	156 (49%)	59 (70%)
** CD4 count (cells/μl), n (%)**			
** <200**	79 (20%)	64 (20%)	15 (18%)
** 200–499**	127 (32%)	97 (31%)	30 (37%)
** ≥500**	190 (48%)	153 (49%)	37 (45%)
HIV viral suppression^b^, n (%)	317 (89%)	254 (88%)	63 (93%)
**Years since ART start, n (%)**			
** <5**	110 (29%)	85 (28%)	25 (34%)
** 5 –<10**	107 (29%)	89 (30%)	18 (24%)
** ≥10**	158 (42%)	127 (42%)	31 (42%)
**Comorbidities, n (%)**	145 (36%)	116 (37%)	29 (35%)
** Arterial hypertension**	86 (22%)	70 (22%)	16 (19%)
** Chronic kidney disease**	46 (12%)	36 (11%)	10 (12%)
** Chronic heart disease**	14 (4%)	11 (4%)	3 (4%)
** Chronic liver disease**	31 (8%)	25 (8%)	6 (7%)
** Diabetes mellitus**	3 (1%)	1 (0.3%)	2 (2.4%)
** Oncological disease**	15 (4%)	12 (4%)	3 (4%)
Co-infection^c^, n (%)	101 (25%)	78 (25%)	23 (27%)
** Parasitic**	20 (5%)	16 (5%)	4 (5%)
** Mycobacteria**	26 (7%)	22 (7%)	4 (5%)
** Bacterial**	28 (7%)	20 (6%)	8 (10%)
** Fungal**	12 (3%)	9 (3%)	3 (4%)
** Viral**	19 (5%)	16 (5%)	3 (4%)
**Laboratory parameters**			
Leucocytes^d^ <4.5x10^9^/L, n (%)	169 (42%)	133 (42%)	36 (43%)
Platelets^d^ >450 x10^9^/L, n (%)	94 (24%)	71 (23%)	23 (27%)
MXD^d^ >20%, n (%)	227 (57%)	187 (59%)	40 (48%)
**MCV (blood analyser), n (%)**			
** Microcytosis**	233 (58%)	179 (57%)	54 (64%)
** Normocytosis**	146 (37%)	124 (39%)	22 (26%)
** Macrocytosis**	21 (5%)	13 (4%)	8 (10%)
**HCT (blood analyser), n (%)**			
** Hypochromia**	327 (82%)	259 (82%)	68 (81%)
** Normochromia**	72 (18%)	56 (18%)	16 (19%)
** Hyperchromia**	1 (0.3%)	1 (0.3%)	0 (0%)

BMI: body mass index; HCT: haematocrit; MCH: mean cell haemoglobin; MCV: mean cell volume; MXD: monocytes-basophils-eosinophils mixed; ZDV: zidovudine. Results are number and percent of those with non-missing data or median and interquantile range (IQR)

^a^Baseline is at the time of inclusion in anaemia study, CD4 window -4/+4 months (from baseline) and VL -6/+6 months

^b^Virally suppressed defined as viral load <100 copies/ml

^c^Parasitic (Malaria, toxoplasma); Mycobacteria (tuberculosis); Bacterial (blood; pneumonia); Fungal infection (candida, crypto); Viral (chronic hepatitis B); Spirochetes (syphilis)

^d^Normal reference ranges: Leucocytes (4.5–11 x10^9^/L); Platelets (150–450 x10^9^/L); MXD (5–10%).

### Anaemia aetiology

**Fig 2:**
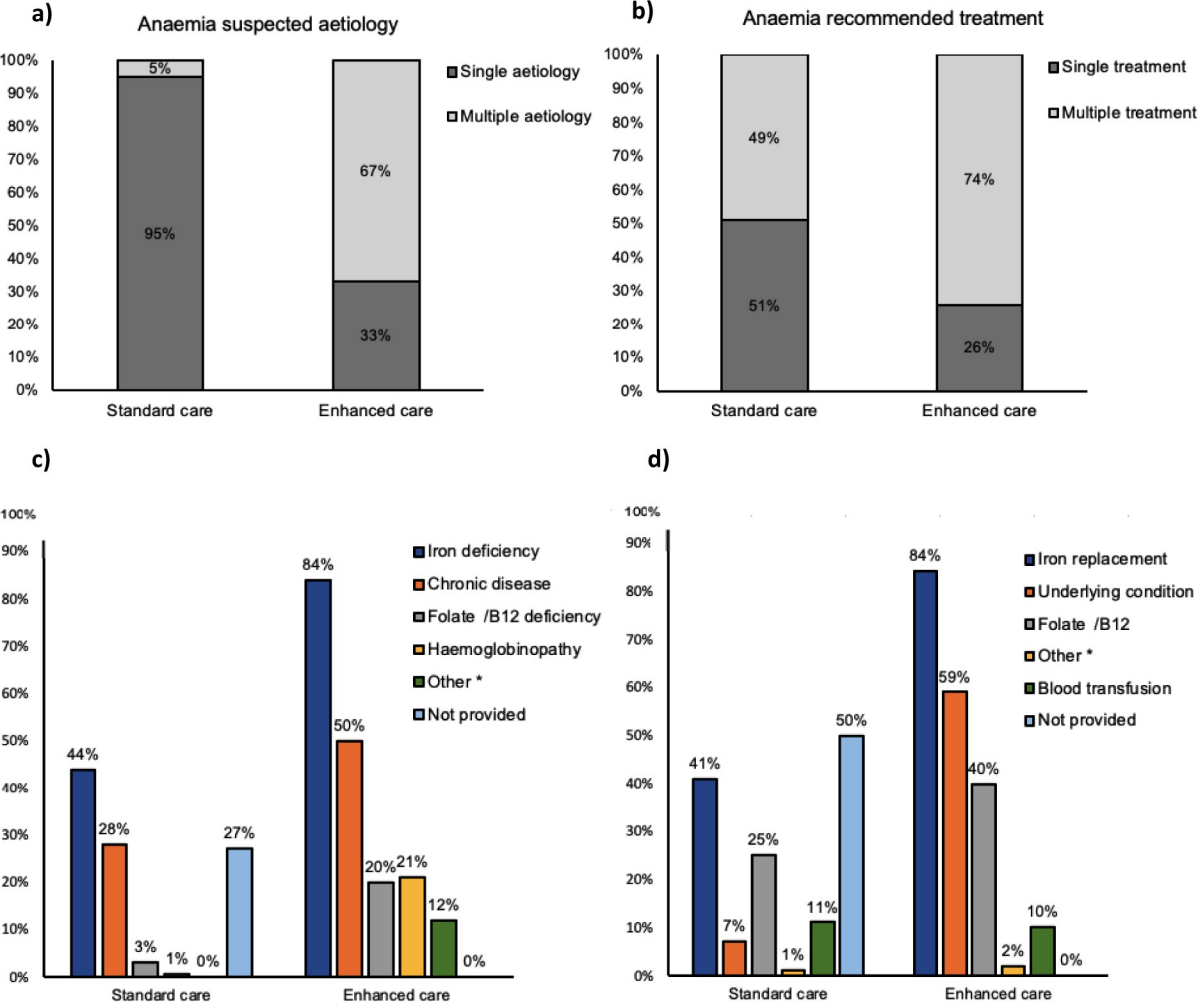
a) Single and multiple aetiologies of anaemia provided by standard care and enhanced care. b) Single and multiple treatment recommendations indicated by standard care and enhanced care. c) Aetiologies of anaemia for standard and enhanced care. d) Treatment recommendations for standard and enhanced care. *Other (graph c) includes bone marrow toxicity and haemolysis. *Other (graph d) includes discontinuation of haematotoxic drug.

In the majority of participants (n = 267; 67%), enhanced care identified multiple aetiologies, which was significantly higher compared to standard care (n = 20;5%); p<0.001 (**Fig 2)**. Among the suspected aetiologies of anaemia identified by enhanced care, iron deficiency was the most frequent aetiology in all participants (n = 337/400; 84%), followed by chronic disease in 199 (50%) participants, folate/vitamin B12 deficit in 78 (20%), haemoglobinopathy in 83 (21%), suspected haemolysis in 37 (9%) and suspected bone marrow suppression in 12 (3%) (**Table 3)**. Suspected types of haemoglobinopathy by PBS interpretation were thalassaemia trait (n = 56; 68%), followed by haemoglobin C (n = 21; 25%) and sickle cell trait (n = 4; 5%). Suspected membranopathy included spherocytosis (n = 1; 1%).

**Table 3:** Suspected anaemia aetiology, treatment recommendation and added clinical value of PBS for moderate and severe anaemia for enhanced care.

	ALL	Moderate	Severe
	N = 400	N = 316	N = 84
**Suspected anaemia aetiology**			
**All (single and multiple aetiologies)**	400 (100%)	316 (100%)	84 (100%)
** Iron deficiency component**	337 (84%)	260 (82%)	77 (92%)
** Chronic disease component**	199 (50%)	169 (53%)	30 (36%)
** Folate and/or B12 deficit component**	78 (20%)	55 (17%)	23 (27%)
Haemoglobinopathy component^1^	83 (21%)	61 (19%)	22 (27%)
** Other**	49 (12%)	40 (13%)	9 (11%)
** Suspected haemolysis**	37 (9%)	30 (9%)	7 (8%)
** Suspected bone marrow suppression**	12 (3%)	10 (3%)	2 (2%)
**Single aetiology**	133 (33%)	108 (34%)	25 (30%)
** Iron deficiency**	76 (19%)	58 (18%)	18 (21%)
** Chronic disease**	44 (11%)	39 (12%)	5 (6%)
** Folate and/or B12 deficiency**	1 (0%)	1 (0%)	0 (0%)
** Other (suspected bone marrow suppression)**	12 (3%)	10 (3%)	2 (2%)
Multiple aetiologies^2^	267 (67%)	208 (66%)	59 (70%)
** Iron deficiency component**	261 (65%)	202 (64%)	59 (70%)
** Chronic disease component**	155 (39%)	130 (41%)	25 (30%)
** Folate and/or B12deficit component**	77 (19%)	54 (17%)	23 (27%)
Haemoglobinopathy component^1^	83 (21%)	61 (19%)	22 (27%)
** Other (suspected haemolysis component)**	37 (9%)	30 (9%)	7 (8%)
**Treatment recommendation**			
**ALL (Single and multiple management)**	400 (100%)	316 (100%)	84 (100%)
** Iron replacement **	337 (84%)	260 (82%)	77 (92%)
Treatment of underlying condition ^3^	235 (59%)	197 (62%)	38 (45%)
Folates and/or B12 replacement ^4^	161 (40%)	119 (38%)	42 (50%)
Other ^5^	9 (2%)	8 (3%)	1 (1%)
** Blood transfusion**	41 (10%)	19 (6%)	22 (26%)
Single management	105 (26%)	90 (28%)	15 (18%)
** Iron replacement **	61 (15%)	51 (16%)	10 (12%)
** Treatment of underlying condition**	43 (11%)	38 (12%)	5 (6%)
** Folates and/or B12 replacement**	1 (0%)	1 (0%)	0 (0%)
Multiple management^2^	295 (74%)	226 (72%)	69 (82%)
** Iron replacement **	276 (69%)	209 (66%)	67 (80%)
** Treatment of underlying condition**	192 (48%)	159 (50%)	33 (39%)
** Folates and/or B12 replacement**	160 (40%)	118 (37%)	42 (50%)
** Other**	9 (2%)	8 (3%)	1 (0%)
** Blood transfusion**	41 (10%)	19 (6%)	22 (26%)
**Added clinical value of the PBS**			
**Added clinical value leading to treatment change**	272 (68%)	206 (65%)	66 (79%)
** Suspected multiple aetiologies**	216 (54%)	166 (53%)	50 (60%)
Folate/B12 deficiency + Iron deficiency ^6^	73 (18%)	53 (17%)	20 (24%)
Iron deficiency + chronic disease ^7^	60 (15%)	52 (16%)	8 (10%)
Haemoglobinopathy + Iron deficiency ^8^ *	83 (21%)	61 (19%)	22 (26%)
** Suspected haemolysis**	4 (1%)	4 (1%)	0 (0%)
** Suspected haemato-oncology disorder**	6 (2%)	2 (0.6%)	4 (5%)
**Suspected bone marrow suppression**	12 (3%)	10 (3%)	2 (2%)
** Microorganisms**	34 (8.5%)	24 (8%)	10 (12%)
** Malaria**	20 (5%)	16 (5%)	4 (5%)
** Bacteraemia/ fungaemia**	14 (3.5%)	8 (3%)	6 (7%)

^1^ Suspected types of haemoglobinopathy were thalassaemia trait (n = 56), haemoglobin C (n = 21), sick cell trait (n = 4) and spherocytosis (n = 1). In all cases, suspected haemoglobinopthy co-existed with iron deficiency anaemia

^2^Participants may have different multiple aetiologies

^3^Includes treatment of an underlying infection, malignancy, chronic disease and haemolysis

^4^Includes treatment of folates/B12 deficiency, suspected haemoglobinopathy and/or haemolysis

^**5**^ Discontinuation of haematotoxic drug

^**6**^ PBS features including macrocytic and hyperchromic RBCs, hypersegmented neutrophils and oval macrocytes (characteristic of folates/B12 deficiency) co-existing with microcytic and hypochromic RBCs, elliptocytosis, dianocytosis, anisopoikilocytosis

^**7**^ Microcytic and hypochromic RBCs, elliptocytosis and dianocytosis co-existing with normocytic RBC and anisopoikilocytosis

^**8**^ Microcytic and hypochromic RBCs, elliptocytosis, dianocytosis, anisopoikilocytosis, coexisting with: a) basophilic stippling, inclusion bodies in RBCs, polychromasia (suspected coexistence of iron deficiency with thalassaemic trait); b)intracellular crystals (suspected coexistence of iron deficiency with Haemoglobin C); c) coexisting normochromic RBCs, sickled cells, schistocytes, polychromasia (suspected coexistence of iron deficiency with sickle cell disease); d) normocytic, hyperchromic RBCs, spherocytes, polychromasia (suspected coexistence of iron deficiency with spherocytosis)

* Please note that 33 cases (26 moderate and 7 severe) of possible suspected haemolysis are included under “haemoglobinopathy + iron deficiency category”.

### Anaemia treatment recommendation

A combination of different treatments was recommended in 295 (74%) participants by enhanced care compared with 196 (49%) by the standard care, p<0.005 **(Fig 2).** In the treatment recommendation arising from enhanced care, iron supplement therapy was the most frequent treatment in 337 (84%) participants, followed by treatment of the underlying condition in 235 (59%), folate/vitamin B12 replacement treatment in 161 (40%), and discontinuation of haematotoxic drugs in 9 (2%). Blood transfusion was advised in 41 (10%) participants (**Table 3)**.

### Added clinical value

Enhanced care based PBS interpretation revealed additional clinically relevant findings in 272 (68%) participants, leading to changes in treatment recommendation. For example, suspected multiple aetiologies of anaemia were found in 216 (54%) instances, suggesting coexistence of iron deficiency and folate or vitamin B12 deficiency in 73 (18%), haemoglobinopathy and iron deficiency in 83 (21%) and iron deficiency and chronic disease anaemia in 60 (15%). Furthermore, findings suggestive of bone marrow suppression were observed in 12 (3%) participants and a possible haemato-oncology disorder was suspected in 6 (2%) participants. Haemolysis was suspected in 4 (1%) cases. Possible haemolysis was considered since no further testing was available for confirmation. PBS showed unexpected findings such as the presence of microorganisms in 34 (8.5%), including malaria in 20 (5%) and bacteraemia or fungaemia in 14 (3.5%) (**Table 3)**. Among 20 cases of malaria, only 7 were detected using a malaria rapid diagnostic test, while the remaining 13 were identified by PBS. PBS morphological characteristics of moderate and severe anaemia are shown in **S1 Table**.

### Suspected anaemia aetiologies and treatment recommendation provided by enhanced care versus standard care

The aetiological diagnosis with standard care was the same as with enhanced care in 158/337 (47%) cases of iron deficiency, 67/199 (34%) of chronic disease and 7/78 (9%) of folate/vitamin B12 deficiency cases. Looking at the discrepancies, of 337 suspected diagnosis of iron deficiency anaemia provided by enhanced care, standard of care indicated the following aetiologies: 75 (22%) unclear, 92 (27%) chronic disease, 9 (3%) folate/B12 deficiency, 2 (1%) haemoglobinopathy and in 75 (22%) standard of care did not indicate a suspected aetiology for anaemia (**Table 4**).

**Table 4:** Suspected aetiological diagnosis and treatment recommendation for moderate and severe anaemia provided by standard and enhanced care.

Suspected aetiology^1^, n(%)														
					**STANDARD**									
					Unclear		Iron deficiency	Chronic disease	Folate/Vitamin B12	Haemoglobinopathy	Other		Total	
E N H A N C E D		Unclear			0 (0)		0 (0)	0 (0)	0 (0)	0 (0)	0 (0)		0	
		Iron deficiency			75(22)		158(47)	92(27)	9 (3)	2(0.6)	1(0.3)		337 (100)	
		Chronic disease			54(27)		72	67(34)	5 (2.5)	0 (0)	1(0.5)		199 (100)	
		Folate/Vitamin B12			18(23)		30(38)	22(28)	7 (9)	0 (0)	1 (1)		78 (100)	
		Haemoglobinopathy			14(17)		47(57)	19(23)	1 (1)	2 (2)	0 (0)		83 (100)	
		Other^2^			13(27)		21 (43)	13 (27)	1 (2)	1 (2)	0 (0)		49 (100)	
		Total			174		328	213	23	5	3		746	
Recommended treatment^1^, n(%)														
			**STANDARD**											
				Not specified		Iron replacement		Underlying condition	Folates/ Vitamin B12	Blood transfusion	Other		Total	
E N H A N C E D	Not specified			0 (0)		0 (0)		0 (0)	0 (0)	0 (0)	0 (0)		0	
	Iron replacement			142		128(38)		18	11	37 (11)	1 (0.3)		337 (100)	
	Underlying condition			116(49)		74(31)		22(9)	3(1)	18(8)	2(1)		235 (100)	
	Folate/Vitamin B12			55 (34)		63 (39)		12	10	20 (12)	1 (0.6)		161 (100)	
	Blood transfusion			0 (0)		3 (7)		3	1 (2)	34 (83)	0 (0)		41 (100)	
	Other^2^			5		2		1 (11)	0	1 (11)	0 (0)		9 (100)	
	Total			318		270		56	25	110	4		783	

^1^ Grey shading shows same diagnosis and recommended treatment by enhanced care and standard care. Values are n (%)

^2^ Other (suspected aetiology) includes haemolysis and bone marrow suppression. Other (recommended treatment) refers to discontinuation of haemato-toxic treatment.

The recommended treatment with standard care was the same as with enhanced care in 128 (38%) of iron deficiency, 22 (9%) of those with underlying condition, 10 (6%) of folate/vitamin B12 and 34 (83%) of blood transfusion. Looking at the discrepancies, of 337 cases in which iron replacement was recommended by enhanced care, standard of care suggested treatment of underlying condition in 18 (5%), folate/B12 replacement in 11 (3%), blood transfusion in 37 (11%), discontinuation of haemato-toxic treatment in 1 (0.3%) and in 142 (42%) no specific treatment was provided. Differences in treatment recommendation between enhanced care and standard care are shown in (**Table 4**).

## Discussion

To the best of our knowledge, this is the first study to determine the suspected aetiologies of moderate and severe anaemia among PLHIV using remote interpretation of PBS in a rural sub-Saharan African setting. The most common causes of anaemia were deficiencies in iron, folate and vitamin B12, chronic disease and haemoglobinopathies. The enhanced care approach–including remote PBS interpretation combined with clinical details and full blood count interpreted by a haematologist–revealed multifactorial anaemia aetiologies in two thirds of all participants compared to only five percent in the standard of care diagnostic workup. This led to changes in treatment recommendation in 68% of participants.

The proportion of iron deficiency anaemia and chronic disease type anaemia among PLHIV in our study is in line with previous studies from other LRS [37–40]. Reasons for the high prevalence of iron deficiency anaemia observed might in part be explained by the high proportion of women in reproductive age (87%) [41] and an inadequate intake of micronutrients as a result of poor-socio-economic status [39, 42].

In our study, enhanced care revealed multiple aetiologies of anaemia in 67% of participants, with the most frequent combination being iron deficiency and chronic disease. Although the complex aetiology of anaemia in PLHIV is known to be multifactorial, there is limited study data on multiple aetiologies of anaemia, particularly in LRS. This is mainly because a full blood count typically captures only the predominant cause of anaemia, which leads to changes in erythrocyte size and haemoglobin content. For instance, the hallmark result for folate or vitamin B12 deficiency is macrocytosis. However, concomitant iron deficiency, which causes microcytosis, may jeopardise the diagnosis of folate or B12 deficiency or vice versa [40]. In these particular scenarios, the PBS can greatly contribute to the aetiological diagnosis of anaemia.

In a study from Malawi, multiple co-existing aetiologies were identified in 95% of the participants with severe anaemia with the most common being unsuppressed HIV infection followed by co-infections [43]. In contrast, in our cohort most of the participants (87%) were virologically supressed on ART and only one quarter presented with co-infections. However, these results should be taken with caution, since extended biochemistry, microbiology and histopathology testing was not available in our setting, contrary to the cohort in Malawi.

The finding that multiple causes of anaemia were more common using PBS with remote interpretation versus standard of care (67% vs 5%) has important treatment implications, as specific diagnosis lead to different treatments [25].

The enhanced care approach provided additional clinical value in two-thirds of cases, allowing changes in treatment recommendation. A prior study in a high-income setting has shown that only one quarter of PBS reviews revealed findings with potential added clinical value [44]. This discrepancy may be attributed to variations in PBS indications, which were not limited to anaemia alone, as well as differences in the population and setting. In our study, unexpected findings such as the presence of microorganisms, features consistent with haemolysis or a possible haemato-oncology disorder made a valuable contribution to patient management. However, in settings with broader diagnostic capabilities, these findings could have been detected and confirmed through alternative tests that were not available in our study.

The main strength of our study is the large, well characterised and prospective clinical cohort and the collection of routine care data in a rural setting in sub-Saharan Africa. Several limitations to this study need to be acknowledged. Firstly, hospitalised PLHIV frequently received blood transfusion by the treating physician, and could therefore not be included, leading to possible selection bias. Secondly, although PBS examination can narrow the most likely aetiology of anaemia, confirmation usually requires a specific test, which in a rural setting is not available, therefore lacking final proof–which on the other hand is the major reason for a study like ours. For instance, suspected haemolysis could not be confirmed in our setting due to limited access to diagnostics, which may have introduced bias into the results. Lastly, morphological interpretation of PBS can be affected by storage time, staining quality and operator skills, which may have played some role on the diagnostic accuracy.

To conclude, anaemia among PLHIV is complex and multifactorial. Especially in virally suppressed PLHIV, adequate diagnosis of causes of anaemia is key to determine appropriate clinical management. Remote interpretation of PBS combined with full blood count results and clinical information might be a useful low-cost diagnostic tool, that can provide insights to the aetiological diagnosis of moderate and severe anaemia and guide specific treatment management among PLHIV in rural LRS.

### The Kilombero and Ulanga Antiretroviral Cohort study group (KIULARCO)

Aschola Asantiel^1^, Farida Bani^1^, Manuel Battegay^2^, Theonestina Byakuzana^1^, Joyce Claud^1^, Adolphina Chale^1^, Elizabeth Dotto^1^, Gideon Francis^3^, Tracy R.Glass^4,5^, Yvonne Haridas^1,3^, Speciosa Hwaya^3^, Aneth V Kalinjuma^1,6,7^, Andrew Katende^1^, Amiri Kayera^2^, Yassin Kisunga^1^, Olivia Kitau^1^, Bernard Kivuma^1^, Thomas Klimkait^6^, Juma Kupewa^1^, Namsifueli J Ley^1^, Ezekiel-Luoga^1^, Jerome Lwali^1^, Swalehe Masoud^1^, Mohammed Mbaruku^1^, Geofrey Mbunda^1^, Josephine Mhina^1^, Slyakus Mlembe^1^, Mengi Mkulila^2^, Margareth Mkusa^2^, Lina Mnunga^2^, Alpha Mninje^2^, Dorcas K Mnzava^1^, Getrud J Mollel^1^, Lilian Moshi^1^, Germana Mossad^2^, Dolores Mpundunga^2^, Athumani Mtandanguo^1^, Elizabeth Mwambashi^2^, Selerine Myeya^1^, Sanula Nahota^1^, Sharifa Nakapala^2^, Regina Ndaki^1^, Robert C. Ndege^1^, Suzan Ngahyoma^3^, Agatha Ngulukila^1^, Alex John Ntamatungiro^1,6,7^, Amina Nyuri^1^, James Okuma^4,5^, Daniel H Paris^4,5^, Martin Rohacek^1,4,5^, Petro Togolani Sabuni^1^, Leila Samson^1^, Elizabeth Senkoro^1^, George Sigalla^1^, Jamali B Siru^1^, Jenifa Tarimo^1^, Juerg Utzinger^4,5^, Fiona Vanobberghen^4,5^, Maja Weisser^1,4,5,8^, John Wigay^1^, Herieth Wilson^1^, Lulu Wilson^1^.

^1^ Interventions and Clinical Trials Department, Ifakara Health Institute, Ifakara, Tanzania

^2^ University Hospital Basel, Basel Switzerland

^3^ St. Francis Referral Hospital, Ifakara, Tanzania

^4^ Swiss Tropical and Public Health Institute, Allschwill, Switzerland

^5^ University of Basel, Basel, Switzerland

^6^ Epidemiology and Biostatistics Department, School of Public Health, Faculty of Health Sciences, University of the Witwatersrand, Johannesburg, South Africa

^7^ Division of Public Health, School of Public Health and Family Medicine, University of Cape Town, South Africa

^8^ Division of Infectious Diseases and Hospital Epidemiology, University Hospital Basel, University of Basel, Switzerland

Maja Weisser is the Research coordinator of the KIULARCO study group

## Supporting information

S1 Fig Study Workflow. **a)** Patients with moderate or severe anaemia had a peripheral blood smear (PBS) performed on the same or the next day. **b)** Ten images of each PBS were taken with an optical microscope with a digital camera attached (under the 100x oil immersion lens). The images were sent together with results from the automated blood analyser and anonymized patient details via a secured online platform to the haematologist. **c)** Within the following 2 weeks, the remote haematologist provided a written report with the suspected aetiology of anaemia and the recommended treatment (enhanced care). **d)** During each follow-up appointment, management was modified according to enhanced care treatment recommendations. Patients with moderate anaemia were scheduled as per standard routine follow-up (12 weeks), whereas those with severe anaemia were scheduled within two weeks.
(DOCX)

S1 Table Blood smear morphological characteristics of moderate and severe anaemia. (DOCX)

S1 Dataset (SAV)

S2 Dataset (XLSX)
