# Supplementary material for: Improving anaemia diagnosis using peripheral blood smear with remote interpretation in adults living with HIV with moderate to severe anaemia: A prospective study nested within the Kilombero and Ulanga antiretroviral cohort
Source: PLoS One. 2023 Oct 19;18(10):e0293084. doi: 10.1371/journal.pone.0293084 (PMC10586595; doi:10.1371/journal.pone.0293084)
Supplement: S1 Fig — a) Patients with moderate or severe anaemia had a peripheral blood smear (PBS) performed on the same or the next day. b) Ten images of each PBS were taken with an optical microscope with a digital camera attached (under the 100x oil immersion lens). The images were sent together with results from the automated blood analyser and anonymized patient details via a secured online platform to the haematologist. c) Within the following 2 weeks, the remote haematologist provided a written report with the suspected aetiology of anaemia and the recommended treatment (enhanced care). d) During each follow-up appointment, management was modified according to enhanced care treatment recommendations. Patients with moderate anaemia were scheduled as per standard routine follow-up (12 weeks), whereas those with severe anaemia were scheduled within two weeks. (DOCX) [file pone.0293084.s001.docx]

**Figure S1: Study Workflow. a)** Patients with moderate or severe anaemia had a peripheral blood smear (PBS) performed on the same or the next day. **b)** Ten images of each PBS were taken with an optical microscope with a digital camera attached (under the 100x oil immersion lens). The images were sent together with results from the automated blood analyser and anonymized patient details via a secured online platform to the haematologist. **c)** Within the following 2 weeks, the remote haematologist provided a written report with the suspected aetiology of anaemia and the recommended treatment (enhanced care). **d)** During each follow-up appointment, management was modified according to enhanced care treatment recommendations. Patients with moderate anaemia were scheduled as per standard routine follow-up (12 weeks), whereas those with severe anaemia were scheduled within two weeks.

0-1 days


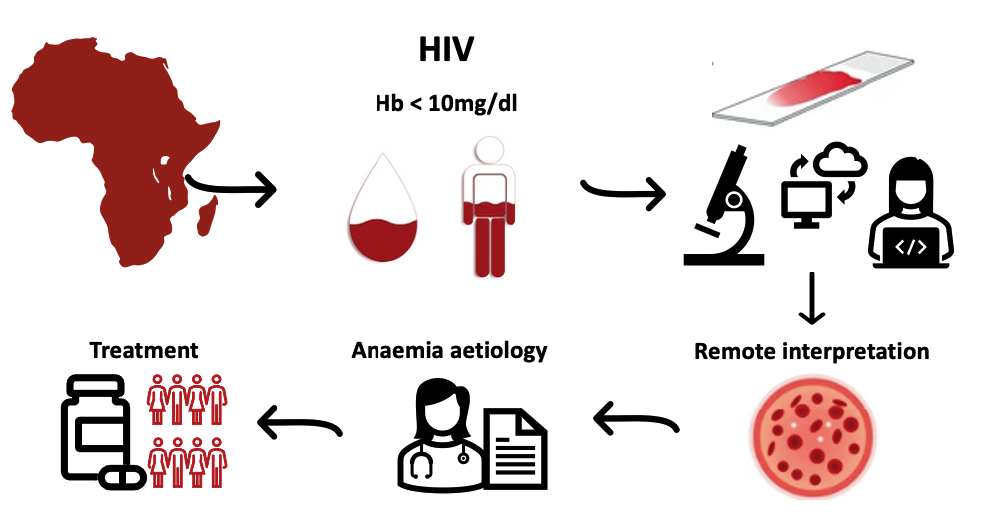


**c)**

**d)**

**b)**

**a)**

≤ 2 weeks ^1^

0-12 weeks ^1^

0-1 days

**^1^** In cases of severe anaemia (Hb <7 g/dl) and/or hospital admission or clinical deterioration, the time to report to the clinician by the haematology team was normally under 24 hours and always within 48 hours. In the aforementioned cases, treatment was initiated immediately after the clinicians received the report.
