# Supplementary material for: Improving anaemia diagnosis using peripheral blood smear with remote interpretation in adults living with HIV with moderate to severe anaemia: A prospective study nested within the Kilombero and Ulanga antiretroviral cohort
Source: PLoS One. 2023 Oct 19;18(10):e0293084. doi: 10.1371/journal.pone.0293084 (PMC10586595; doi:10.1371/journal.pone.0293084)
Supplement: S1 Table — (DOCX) [file pone.0293084.s002.docx]

**Table S1** Blood smear morphological characteristics of moderate and severe anaemia.

|  | ALL  N=400  n (%) | Moderate  N=316  n (%) | Severe  N=84  n (%) |
| --- | --- | --- | --- |
| Monomorphic  Microcytosis/ Hypochromia  Normocytosis/ Hypochromia  Normocytosis/ Normochromia  Macrocytosis/ Normochromia  Microcytosis/ Normochromia  Macrocytosis/ Hypochromia  Dimorphic  Micro-normocytosis/ Hypo-normochromia  Micro-macrocytosis/ Hypo-normochromia  Normo-macrocytosis/ Hypo-normochromia | 291 (73%)  164 (41%)  86 (22%)  37 (9%)  5 (1%)  4 (1%)  1 (0.3%)  109 (27%)  60 (15%)  38 (10%)  5 (1%) | 231 (73%)  123 (39%)  73 (23%)  30 (10%)  5 (2%)  2 (1%)  1 (0.3%)  85 (27%)  51 (16%)  27 (9%)  4 (1%) | 60 (71%)  41 (49%)  13 (16%)  7 (8%)  0 (0%)  2 (2%)  0 (0%)  24 (29%)  9 (11%)  11 (13%)  1 (1%) |
| Red blood cells:  Acanthocytes  Dacriocytes  Drepanocytes  Elliptocytes  Schistocytes  Spherocytes  Stomatocytes  Dianocytes  Echinocytes  Poikilocytosis  Anisocytosis  Anisopoikilocytosis  Macroovalocytes  Irregular distribution of haemoglobin  White blood cells:  Normal characteristics  Hypersegmented neutrophils  Hyposegmented neutrophils  Internuclear bridge  Toxic granulation of neutrophils  Atypical lymphocytes  Lymphocytoblast  Platelets:  Normal characteristics  Anisothrombia  Macro platelets | 12 (3%)  24 (6%)  5 (1%)  193 (48%)  57 (14%)  7 (2%)  42 (11%)  215 (54%)  40 (10%)  13 (3%)  66 (17%)  250 (63%)  92 (23%)  62 (16%)  141 (35%)  75 (19%)  52 (13%)  6 (2%)  18 (5%)  10 (3%)  5 (1%)  197 (49%)  138 (35%)  21 (5%) | 8 (3%)  19 (6%)  4 (1%)  151 (48%)  39 (12%)  4 (1%)  35 (11%)  168 (43%)  34 (11%)  11 (4%)  50 (16%)  193 (61%)  70 (22%)  45 (14%)  106 (34%)  50 (15%)  34 (11%)  5 (2%)  11 (4%)  5 (2%)  3 (1%)  152 (48%)  104 (33%)  19 (6%) | 4 (5%)  5 (6%)  1 (1%)  42 (50%)  18 (21%)  3 (4%)  7 (8%)  47 (56%)  6 (7%)  2 (2%)  16 (19%)  57 (68%)  22 (26%)  15 (20%)  35 (42%)  25 (30%)  18 (21%)  1 (1%)  7 (8%)  5 (6%)  2 (2%)  45 (54%)  34 (41%)  2 (2%) |
| Microrganisms  Malaria parasites  Bacterial/ fungal forms | 34 (8.5%)  20 (5%)  14 (3.5%) | 24 (8%)  16 (5%)  8 (3%) | 10 (12%)  4 (5%)  6 (7%) |
